# Insertion sequences in *mgrB* and mutations in two-component system genes confer high polymyxin resistance to carbapenem-resistant *Enterobacter cloacae* complex strains

**DOI:** 10.3389/fmicb.2025.1553148

**Published:** 2025-03-17

**Authors:** Jiming Wu, Jisheng Zhang, Jianmin Wang, Jin Wang, Xushan Liang, Chunli Wei, Wenzhang Long, Yang Yang, Yuhui Chen, Mingjing Liao, Youtao Liang, Kaixin Yu, Xiaoli Zhang

**Affiliations:** ^1^Department of Microbiology, Yongchuan Hospital of Chongqing Medical University, Chongqing, China; ^2^Department of Pathogenic Biology, Basic Medicine of Jiamusi University, Jiamusi, China

**Keywords:** *Enterobacter cloacae* complex, *mcr-9*, polymyxin, CRECC, epidemiology, antibiotic resistance

## Abstract

Due to the complexity of identifying the *Enterobacter cloacae* complex (ECC) at the species level, little is known about the distribution of carbapenem-resistant ECC (CRECC). Plasmid-mediated *mcr* family genes are significant contributors to polymyxin resistance. The emergence of the *mcr-9* gene has further complicated the landscape of polymyxin resistance in CRECC. Our study aimed to ascertain the prevalence of CRECC and the *mcr-9* gene, and to elucidate the mechanisms underlying high-level resistance to polymyxin B (PB). In this study, we collected 212 non-replicating ECC strains, identifying 38 CRECC strains (17.9%, 38/212) and *Enterobacter hormaechei* (71.1%, 27/38) as the predominant endemic strains. Among these, 10 CRECC strains (36.3%, 10/38) were found to harbor the *mcr-9* gene. Interestingly, the presence of *mcr-9* did not significantly impact PB resistance or impose a fitness cost. While overexpression of *mcr-9* can enhance PB resistance within a certain range and may incur fitness costs, it does not result in high-level PB resistance. The PB resistance of 17 CRECC strains was notably increased (from 16 to 128 mg/L), accompanied by mutations in the *phoP*/*Q* and *mgrB* genes. Notably, two novel insertion sequences, IS*5D* and IS*1X2*, were discovered within the *mgrB* gene. The inactivation of *mgrB* results in the loss of its negative regulatory effect on the two-component system. Protein structure predictions indicated that mutations in *phoQ* primarily affect the phosphatase (HAMP) and histidine kinase domains. This research significantly expands our comprehension of the complexities of PB resistance, highlighting the multifactorial nature of antibiotic resistance mechanisms.

## Introduction

Carbapenem antibiotics are a key treatment strategy for Gram-negative enterobacterial infections, and the prevalence of carbapenem-resistant Enterobacterales (CRE) has been progressively increasing due to the extensive and long-term use of antibiotics (Yoon et al., 2019; Lee et al., 2022; Puljko et al., 2024). Among them, carbapenem-resistant *Enterobacter cloacae* complex (CRECC) is the third most common pathogen, often causing serious infections (Zhang et al., 2018; Girlich et al., 2021). CRECC frequently exhibits multidrug resistance, forcing us to reintroduce polymyxins to combat these difficult-to-treat bacteria (Li et al., 2006). Polymyxin B (PB), belonging to the polymyxin family along with polymyxin E, is considered as a last resort against multi-drug resistant Enterobacterales (Nang et al., 2021). However, there has been an alarming rise in global reports of PB resistance, posing a serious public health concern (Zhang et al., 2021; Liao et al., 2022). *Enterobacter cloacae* complex (ECC) is a complex community composed of multiple species, including *E. hormaechei*, *E. asburiae*, *E. kobei* and *E. cloacae* etc. The members of ECC are difficult to distinguish using the conventional phenotypic/biochemical or mass spectrometry identification methods available in clinical laboratories (Garinet et al., 2018; Girlich et al., 2021). Therefore, the prevalence trend of CRECC at species level still needs to be investigated.

Furthermore, previous studies have demonstrated that the pressure of antibiotics creates resistance to PB, leading to an increased risk of clinical treatment failure (Huang et al., 2020; Li et al., 2021). While most epidemiological reports primarily focus on the inherent resistance of carbapenem-resistant Enterobacterales to PB prior to exposure (Zhang et al., 2021), it is crucial to investigate cases where high-level resistance to polymyxins emerges in strains after exposure (Zhu et al., 2019; Wu et al., 2023). The resistance mechanisms in *Enterobacter* for PB are categorized into chromosomally mediated intrinsic resistance and plasmid-mediated acquired resistance (Poirel et al., 2017; Choi et al., 2020). Intrinsic drug resistance is primarily attributed to the PhoPQ two-component system and its negative regulator MgrB, which modulate downstream *arnBCADTEF* expression and subsequently mediate L-Ara4N modification of lipid A (Nang et al., 2021). While the PmrAB two-component system has been implicated in regulating polymyxin resistance in *Klebsiella pneumoniae*, studies have reported no impact of *pmrAB* on PB resistance within ECC (Doijad et al., 2023). These findings highlight distinct mechanisms of PB resistance across different bacterial species. Plasmid-mediated polymyxin resistance predominantly involves mobile colistin-resistant genes, including *mcr-9* gene with three subtypes (Li et al., 2020; Zelendova et al., 2023). Previous investigations suggest that *mcr-9* is more likely to be acquired and stably inherited by CRE strains; furthermore, there have been reports linking epidemic highly virulent clones carrying both *mcr-9* and carbapenemase genes with *E. hormaechei* infections (Xu et al., 2022b; Zhou et al., 2022). However, further studies are needed to elucidate the specific effects of *mcr-9* on PB resistance and its association with patient clinical outcomes or potential outbreaks posing public health concerns—particularly within widespread *mcr-9*-carrying ECC isolates (Liao et al., 2022; Xu et al., 2022a).

Therefore, a comprehensive analysis was conducted on the prevalence of CRECC and *mcr-9* in clinical ECC isolates. The impact of *mcr-9* and its overexpression on both PB resistance and growth of ECC strains were investigated. Moreover, an *in vitro* induction approach was employed to elucidate the underlying mechanism behind the development of high-level PB resistance in CRECC.

## Materials and methods

### Bacterial isolates, identification and drug susceptibility testing

From December 2018 to April 2023, a total of 212 non-repetitive isolates of ECC were collected from clinical specimens at a teaching hospital (Supplementary Table S2). Identification was conducted using the VITEK-2 automated microbiological analyzer and MALDI-TOF MS (Bruker, Billerica, MA, United States). Based on the results of clinical drug sensitivity testing, 38 CRECC isolates were identified. The minimum inhibitory concentration (MIC) of PB was determined using the broth microdilution method with strain ATCC25922 as the quality control strain. Each MIC determination was repeated three times following CLSI (Clinical and Laboratory Standards Institute) guidelines (Satlin et al., 2020).

### Whole-genome sequencing, core genome MLST and cg-SNP

WGS was performed on 212 ECC isolates using Illumina HiSeq PE150 (Illumina, San Diego, CA, United States), and 10 CRECC isolates carrying *mcr-9* for further long-read sequencing (Oxford Nanopore, Oxford, United Kingdom). Long-read sequencing assembly was performed using unicycler v0.4.8 and was corrected using pilon v1.22 (Walker et al., 2014; Wick et al., 2017). All strains contained 3,048 core genomes according to the reference genome (GenBank accession no. CP010377). The visualization of cg-MLST results is based on the previous methodology (Stamatakis, 2014). The results of core genome single nucleotide polymorphism (cg-SNP) were visualized using ggtree after building the evolutionary tree with FastMe (Lefort et al., 2015; Xie et al., 2023). FastANI was employed to rapidly calculate ANI and classify ECC species (Jain et al., 2018).

### *In vitro* polymyxin B resistant induction by exposure to polymyxin B

In order to obtain CRECC strains with high-level resistance to PB through induction, we made certain modifications based on previous studies and conducted a 7-day *in vitro* induction experiment (Wu et al., 2023). Initially, overnight cultures of 36 isolates of CRECC were incubated in 1 mg/L PB for 24 h. Subsequently, 10 μL aliquot of the overnight culture was inoculated onto blood plates and incubated at 37°C for 18 h to assess growth continuation. Simultaneously, a volume of 20 μL from the overnight culture was inoculated into fresh LB broth (4 mL) with doubled PB concentration. Finally, the MIC of induced strains toward PB was determined using the broth microdilution method.

### Detection of mutations in genes related to polymyxin resistance

All successfully induced CRECC isolates with high levels of PB resistance were detected by PCR for mutations in related genes. Genomic DNA was extracted using TIANamp bacterial DNA kit (Tiangen Biotechnology, Beijing, China) according to the manufacturer’s recommendations. The collected DNA was detected by gel electrophoresis using 1.2% agarose (Invitrogen) in 0.5 × Tris Borate EDTA running buffer (BIO-RAD) and quantified by ND 2000 (NanoDrop Technologies). Sanger sequencing of the positive amplification products was performed at Shanghai Shenggong (Shangai, China).

### Conjugation assay

According to the method described previously, the bonding experiment was conducted using the membrane bonding method (Gong et al., 2018). The donor (CRECC68) and recipient culture (*E. coli* EC600) were mixed in a 1:3 ratio in Luria-Bertani (LB) broth. The mixture was inoculated in a filter membrane and incubated at 37 ° C for 24 h. Meropenem (1 mg/L) and rifampicin (600 mg/L) were added to the Muller-Hinton agar plate to screen the transconjugants. The presence of transconjugants were confirmed by VITEK-2 compact system and PCR.

### Construction of the *mcr-9.1* deletion mutant

The upper and downstream homologous recombination arm of the *mcr-9.1* gene was amplified from the CRECC68 genome by superfidelity DNA polymerase; the apramycin resistance gene (*apr*) was amplified from the pUC57-*apr* plasmid; the upper and downstream homologous recombination arm of the *mcr-9.1* gene and *apr* by fusion PCR to generate the target fragment Δ*mcr-9.1*, which was cloned into the suicide plasmid pCVD442 to obtain the target plasmid pCVD442-Δ*mcr-9.1*. By electroporation, pCVD442-Δ*mcr-9.1* was transferred to *E. coli* β2155 to obtain the donor bacteria β2155/pCVD442-Δ*mcr-9.1*. β2155/pCVD442-Δ*mcr-9.1* was used as donor to conjugate with the receptor of *E. cloacae*. Several Ecl/pCVD442-Δ*mcr-9.1* clone solutions were spread on LB plates containing 10% sucrose and grown until the single clones were formed. The clones with the *mcr-9.1* gene knockout were selected by PCR screening and named CRECC68-Δ*mcr-9.1*. PB susceptibility testing was subsequently performed on CRECC68-Δ*mcr-9.1* to verify the function of the *mcr-9.1* gene. The primers used in this experiment are shown in the Supplementary Table S1.

### Construction of *mcr-9.2* expression strain

The full length of the *mcr-9.2* gene was amplified from the genomic DNA of CRECC414 isolate using PCR. The PCR products and pET-28a plasmid were enzymatically digested at 37°C for 3 h. The purified target gene and pET-28a plasmid were ligated using T4 DNA ligase, followed by transformation into DH5α recipient cells and spreading on LB plates containing kanamycin (100 μg/mL). Monoclonal strains were selected the next day and verified through PCR and agarose gel electrophoresis. After successful verification, the PB MIC value of recombinant bacterium DH5α + pET-28a + *mcr-9.2* was determined.

### Growth curves

The growth curve of the strain was determined by measuring the absorbance at 600 nm using an Multifunctional Microplate Reader (Thermo Scientific) within a 24-h period. The strain culture was inoculated in LB medium broth at a ratio of 1:100 and incubated at 37°C with agitation at 180 rpm. At the third hour, IPTG (1 μM) was added to induce the expression of *mcr-9* in the recombinant strain. The experiment was repeated three times.

### Outer membrane permeability analysis

The N-phenyl-1-naphthylamine (NPN) uptake test was employed to assess the permeability of the outer membrane, following previously established protocols (Burow et al., 2009; Lu et al., 2022). In brief, bacterial samples were collected during the logarithmic growth phase. Cultures of *E. coli* harboring the recombinant plasmid pET-28a + *mcr-9.2* and control strains were centrifuged at 1,000 g for 5 min. Subsequently, cells were washed thrice with PBS and resuspended to an optical density (OD600) of 0.5. The bacterial suspension was thoroughly mixed with NPN at a final concentration of 10 μM. After incubation at room temperature for 10 min, cell suspensions were analyzed using Multifunctional Microplate Reader (Thermo Scientific). Fluorescence emission was measured at an excitation wavelength of 350 nm and an emission wavelength of 450 nm.

## Results

### Prevalence of CRECC

**Figure 1:**
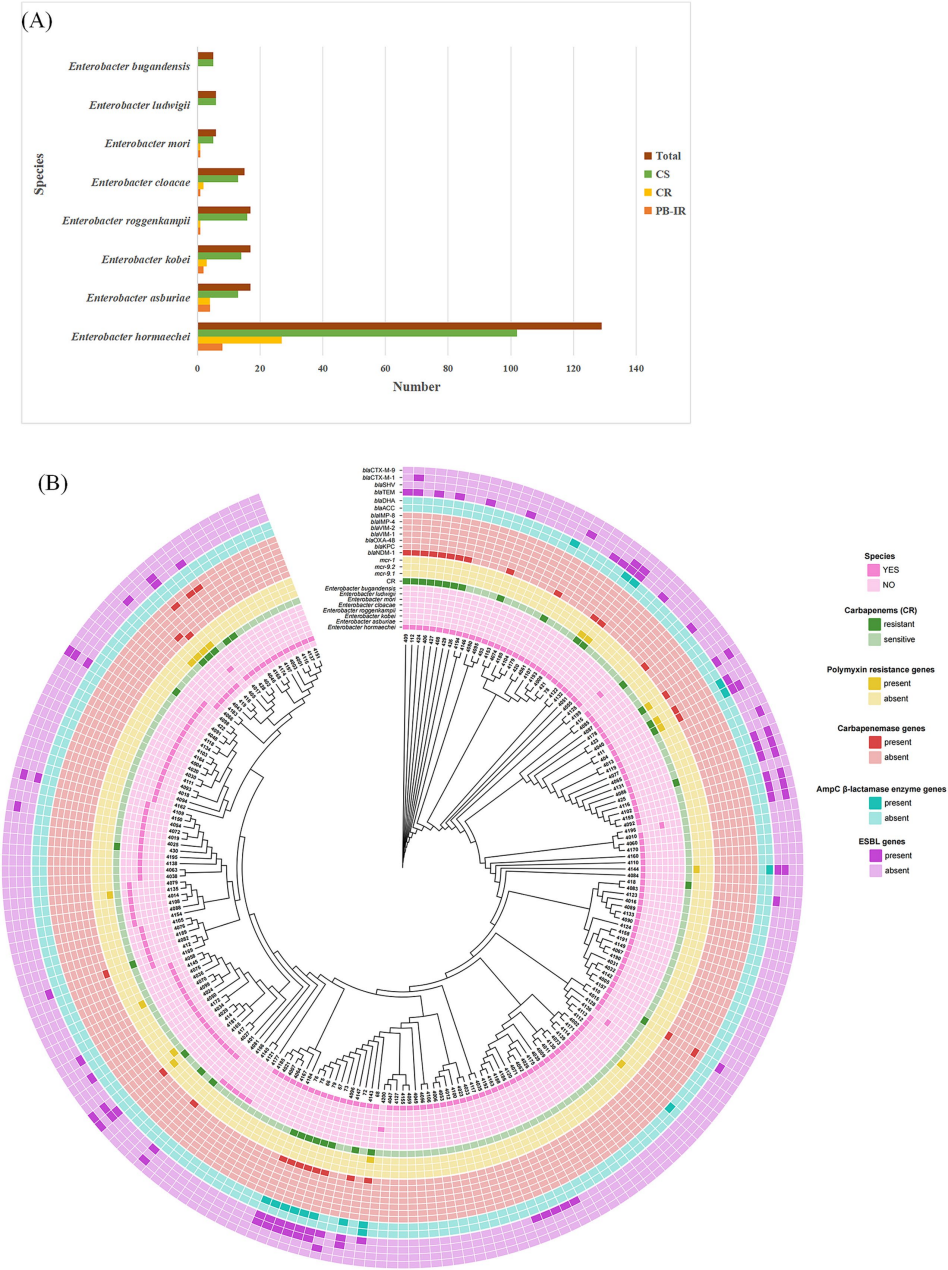
Epidemiological background of isolates. **(A)** Distribution characteristics of 212 isolates of *Enterobacter cloacae* complex at species level. CS, carbapenem sensitive; CR, carbapenem resistant; PB-IR, Polymyxin B (PB) induced resistance. **(B)** Phylogenetic analysis and characterization of drug resistance gene carriage were conducted in a cohort of 212 ECC strains.

We collected 212 non-duplicate clinical strains of the ECC, including 38 (17.9%) CRECC and 174 (82.1%) carbapenem-sensitive ECC (CSECC) (Figure 1A; Supplementary Table S2). The prevalence of carbapenem resistance among the ECC strains was as follows: *E. hormaechei* (20.9%, 27/129), *E. asburiae* (23.5%, 4/17), *E. kobei* (17.6%, 3/17), *E. roggenkampii* (5.9%, 1/17), *E. cloacae* (13.3%, 2/15), and *E. mori* (16.7%, 1/6). All isolates of *E. ludwigii* and *E. bugandensis* exhibited susceptibility to carbapenems. The *mcr-9* carriage rate among ECC strains was 7.5% (16/212), with the following distribution: *E. hormaechei* (*n* = 8), *E. kobei* (*n* = 3), *E. asburiae* (*n* = 3), *E. cloacae* (*n* = 1), and *E. ludwigii* (*n* = 1) (Figure 1B). The metallo-carbapenemase gene *bla*_NDM-1_ was found in 76.3% (29/38) of CRECC strains (Figure 1B). Additionally, Figure 1B depicts the specific carriage patterns of AmpC β-lactamase and ESBL genes. Notably, *mcr-1*, a gene associated with polymyxin resistance, was not detected.

A total of 38 non-duplicate clinical strains of CRECC were collected. Of these, 10 carried the *mcr-9* gene, with 9 containing *mcr-9.1* and 1 containing *mcr-9.2* (Table 1; Figure 1B). The 38 CRECC isolates were classified into six species, with *E. hormaechei* being the predominant strain (71.1%, 27/38). Other species included *E. asburiae* (10.5%, 4/38), *E. kobei* (7.9%, 3/38), *E. roggenkampii* (2.6%, 1/38), *E. cloacae* (5.3%, 2/38), and *E. mori* (2.6%, 1/38). The main clinical sources of CRECC strains were blood (44.7%, 17/38) and urine (18.4%, 7/38). The *mcr-9* gene was present in 26.3% (10/38) of CRECC strains, compared to 3.4% (6/174) in CSECC. CRECC strains are often accompanied by *mcr-9* gene, which is consistent with relevant studies (Xu et al., 2022a; Zhou et al., 2022; Zeng et al., 2023). The high prevalence of the *mcr-9* gene in CRECC strains had been concerning due to its association with high levels of PB resistance, presenting a significant public health issue.

**Table 1:** Characteristics of 38 CRECC strains.

Isolates	*mcr-9* ^a^	Species	Sample type	PB MIC^c^ (mg/L)
CRECC66	–^b^	*Enterobacter hormaechei*	Sputum	2
CRECC76	–	*Enterobacter hormaechei*	Sputum	4
CRECC68	*mcr-9.1*	*Enterobacter hormaechei*	Sputum	4
CRECC410	–	*Enterobacter hormaechei*	Bronchoalveolar lavage fluid	2
CRECC67	–	*Enterobacter hormaechei*	Sputum	2
CRECC72	–	*Enterobacter hormaechei*	Sputum	2
CRECC73	–	*Enterobacter hormaechei*	Sputum	4
CRECC75	–	*Enterobacter hormaechei*	Urine	4
CRECC422	–	*Enterobacter roggenkampii*	Urine	1
CRECC430	–	*Enterobacter cloacae*	Bile	0.5
CRECC401	–	*Enterobacter kobei*	Bronchoalveolar lavage fluid	2
CRECC402	–	*Enterobacter asburiae*	Sputum	2
CRECC412	–	*Enterobacter mori*	Blood	2
CRECC414	*mcr-9.2*	*Enterobacter kobei*	Sputum	4
CRECC416	*mcr-9.1*	*Enterobacter asburiae*	Urine	2
CRECC419	*mcr-9.1*	*Enterobacter asburiae*	Drainage fluid	2
CRECC428	–	*Enterobacter asburiae*	Sputum	1
CRECC78	*mcr-9.1*	*Enterobacter hormaechei*	Bile	2
CRECC112	–	*Enterobacter hormaechei*	Sputum	1
CRECC408	–	*Enterobacter hormaechei*	Sputum	2
CRECC420	–	*Enterobacter hormaechei*	Sputum	1
CRECC421	*mcr-9.1*	*Enterobacter hormaechei*	Bronchoalveolar lavage fluid	1
CRECC423	*mcr-9.1*	*Enterobacter hormaechei*	Sputum	1
CRECC424	–	*Enterobacter hormaechei*	Urine	1
CRECC425	–	*Enterobacter hormaechei*	Sputum	1
CRECC426	–	*Enterobacter hormaechei*	Drainage fluid	1
CRECC427	–	*Enterobacter hormaechei*	Sputum	2
CRECC403	–	*Enterobacter hormaechei*	Blood	2
CRECC406	–	*Enterobacter hormaechei*	Sputum	2
CRECC409	–	*Enterobacter hormaechei*	Urine	2
CRECC411	*mcr-9.1*	*Enterobacter hormaechei*	Urine	1
CRECC415	–	*Enterobacter hormaechei*	Blood	2
CRECC429	–	*Enterobacter hormaechei*	Drainage fluid	0.5
CRECC404	*mcr-9.1*	*Enterobacter hormaechei*	Drainage fluid	1
CRECC79	–	*Enterobacter hormaechei*	Sputum	2
CRECC418	–	*Enterobacter hormaechei*	Bile	0.5
CRECC405	*mcr-9.1*	*Enterobacter cloacae*	Bronchoalveolar lavage fluid	128
CRECC417	–	*Enterobacter kobei*	Urine	32

a *mcr-9 gene carrying status.*

b Not present.

c Minimum inhibitory concentration of polymyxin B (PB).

### Effect of *mcr-9* carriage or overexpression on PB resistance

To investigate the impact of *mcr-9* on PB resistance in clinical strains, we performed gene knockout using homologous recombination and successfully generated the CRECC68-Δ*mcr-9.1* deletion mutant. The PB MIC for wild-type CRECC68 strain was 4 mg/L, whereas the MIC (PB) for CRECC68-Δ*mcr-9.1* decreased by half compared to the wild-type strain (Table 2). Considering the complexity of clinical strain backgrounds, we conducted conjugation and cloning experiments separately. The transconjugant J68 containing *mcr-9.1* gene was obtained by conjugating the plasmid carrying *mcr-9.1* to EC600. Additionally, we cloned the *mcr-9.2* gene into pET-28a vector to construct recombinant strain DH5α + pET-28a + *mcr-9.2*. PB MICs were determined using microbroth dilution method for EC600, J68, DH5α, DH5α + pET-28a, and DH5α + pET-28a + *mcr-9.2* strains. Our results showed that all strains were sensitive to PB, with no significant differences in MIC between EC600 and J68 in the conjugation experiment (Table 2). Similarly, no significant differences in PB MICs were observed among DH5α, DH5α + pET-28a, and DH5α + pET-28a + *mcr-9.2* strains. However, upon IPTG induction for high-level expression of *mcr-9.2* in BL21 (DE3)pLysS+pET-28a + *mcr-9.2* strains, we observed a doubling of the MIC compared to control strains without IPTG. Overall, our findings suggest that both *mcr-9.1* and *mcr-9.2* contribute minimally to PB resistance, indicating that *mcr-9* does not confer high levels of resistance.

**Table 2:** Plasmids and strains used in this study.

Strains	Description	PB MIC^a^ (mg/L)
CRECC68	WT With *mcr-9.1* gene	4
CRECC68-Δ*mcr-9.1*	Without *mcr-9.1* gene	2
EC600	Receptor strain for conjugation experiment	1
J68 (HI2A-IncN-*mcr-9.1*)	EC600 carrying HI2A-IncN plasmid with *mcr-9.1*	2
DH5α	Without plasmid	2
DH5α + pET-28a	DH5α carrying pET-28a plasmid	2
DH5α + pET-28a + *mcr-9.2*	DH5α carrying pET-28a plasmid with *mcr-9.2*	2
BL21(DE3)pLysS	Without plasmid	2
BL21(DE3)pLysS+pET28a	BL21(DE3)pLysS carrying pET-28a plasmid	2
BL21(DE3)pLysS+pET28a (IPTG)	BL21(DE3)pLysS carrying pET-28a plasmid and is induced by IPTG	2
BL21(DE3)pLysS+pET-28a + *mcr-9.2*	BL21(DE3)pLysS carrying pET-28a plasmid with *mcr-9.2*	2
BL21(DE3)pLysS+pET-28a + *mcr-9.2* (IPTG)	BL21(DE3)pLysS carrying pET-28a plasmid with *mcr-9.2* and is induced by IPTG	4
ATCC25922	Quality control strain	2

a Minimum inhibitory concentration of polymyxin B (PB).

### Analysis of bacterial tolerance in monocarriers and overexpression states of the *mcr-9* gene

**Figure 2:**
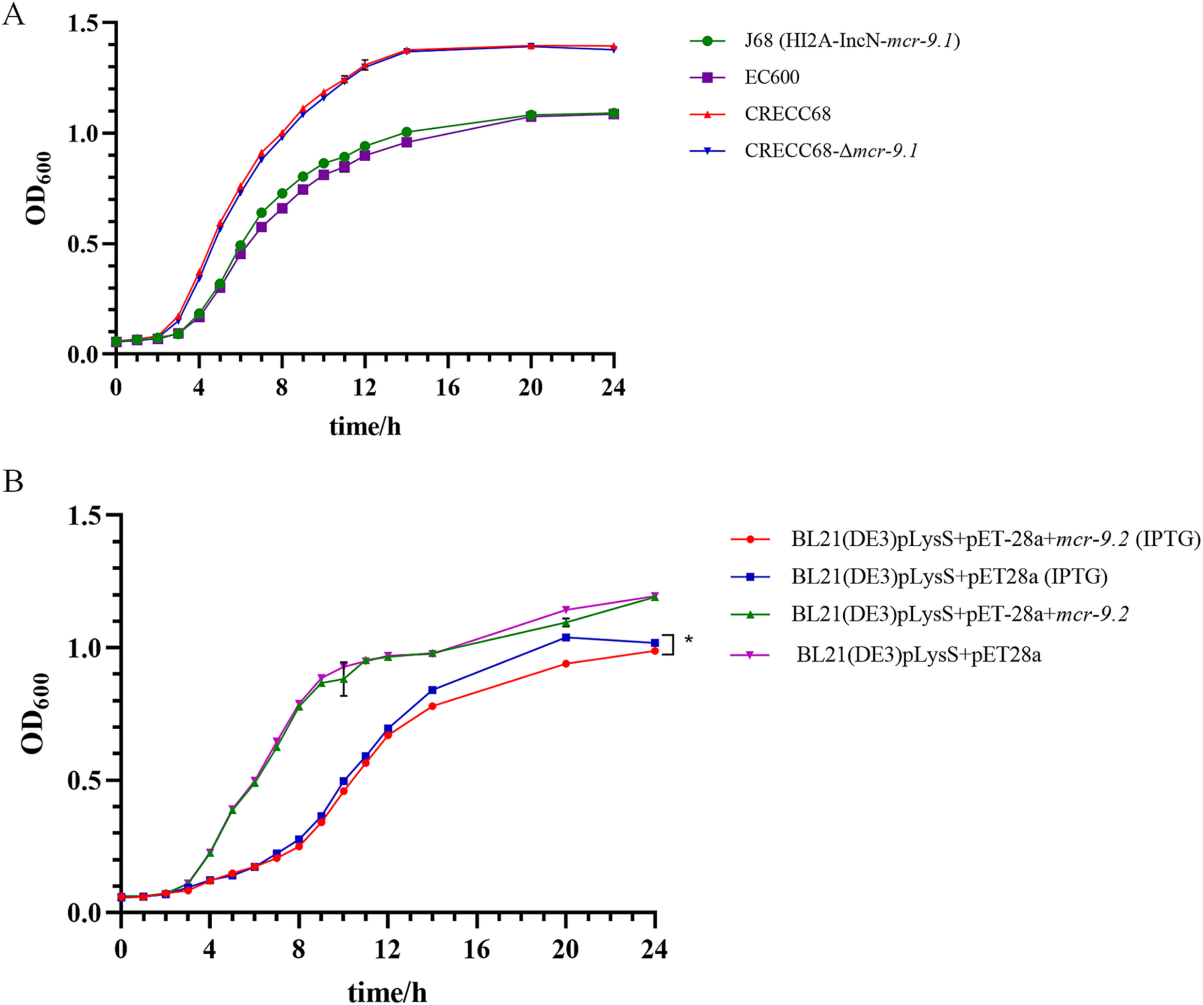
The influence of carrying *mcr-9* and overexpression of *mcr-9* on the growth of ECC. **(A)** Growth curves of transconjugant J68, receptor EC600, wild-type CRECC68 and *mcr-9.1* deletion mutant strain CREC68-Δ*mcr-9.1*. **(B)** Growth curves of *Escherichia coli* BL21(DE3) pLysS harboring empty vector pET-28a or pET-28a + *mcr-9.2* with or without 1 mM IPTG induction. The data shows the mean ± SD of three independent replicates. Statistical significance was determined via a two-way ANOVA; *p ≤ 0.05.

To assess the fitness cost associated with the *mcr-9* gene, we conducted a growth curve analysis. The growth rate of J68 was comparable to that of EC600, and no significant difference was observed between CRECC68 and CRECC68-Δ*mcr-9.1* (Figure 2A). Similarly, the growth of BL21 (DE3)pLysS+pET-28a + *mcr-9.2* was consistent with that of BL21 (DE3)pLysS+pET-28a (Figure 2B). In addition, the high expression of *mcr-9.2* was induced by IPTG, and the bacterial growth was monitored. The growth of BL21(DE3)pLysS+pET-28a + *mcr-9.2*(IPTG) with high expression of *mcr-9.2* gene was slower compared to BL21(DE3)pLysS+pET-28a(IPTG) (Figure 2B). These results indicate that overexpression of *mcr-9.2* incurs a fitness cost.

### Overexpression of *mcr-9* increased the permeability of bacterial outer membrane

**Figure 3:**
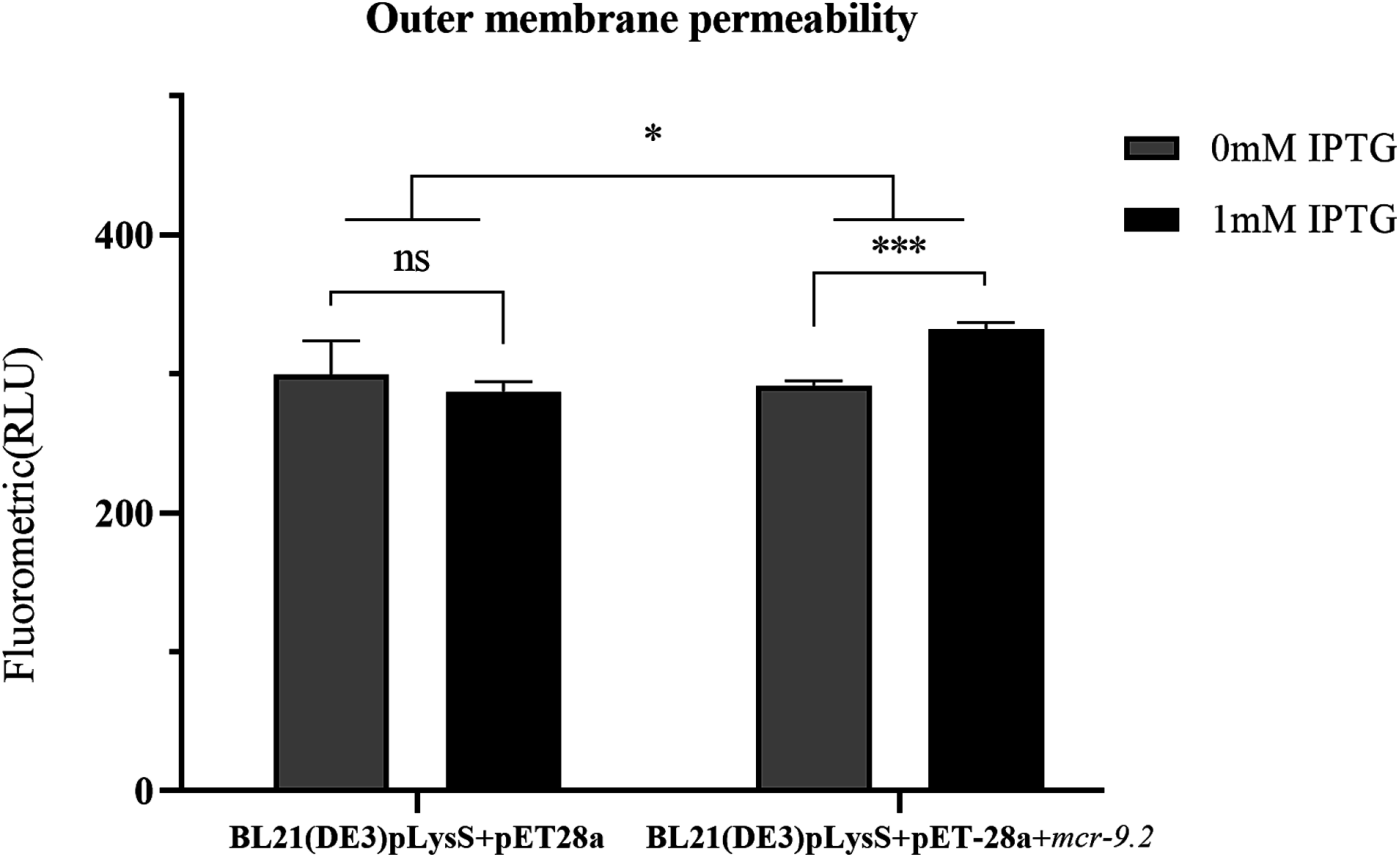
Effect of *mcr-9.2* expression on membrane permeability. Outer membrane permeability of *Escherichia coli* BL21(DE3) pLysS harboring empty vector pET-28a or pET-28a + *mcr-9.2* with or without 1 mM IPTG induction. The asterisks indicate statistical significance at different levels by paired *t*-test: *p ≤ 0.05; ***p ≤ 0.001; ns, not significant.

NPN is a hydrophobic fluorescent probe that can integrate into cell membranes (Lee and Je, 2013). Its fluorescence intensity increases in hydrophobic environments, making it a useful indicator for assessing membrane permeability (Lu et al., 2022). Therefore, we determined the permeability of the outer membrane by NPN uptake test. The fluorescence intensity of BL21(DE3)pLysS+pET-28a + *mcr-9.2* significantly increased with 1 mM IPTG compared to 0 mM IPTG (Figure 3). In contrast, there was no significant difference in the fluorescence intensity of the control group BL21(DE3)pLysS+pET-28a at 1 mM IPTG versus 0 mM IPTG (Figure 3). Thus, overexpression of *mcr-9.2* in the presence of IPTG leads to a significant increase in outer membrane permeability (Figure 3).

### PB induction assay and antibiotic susceptibility experiments

**Figure 4:**
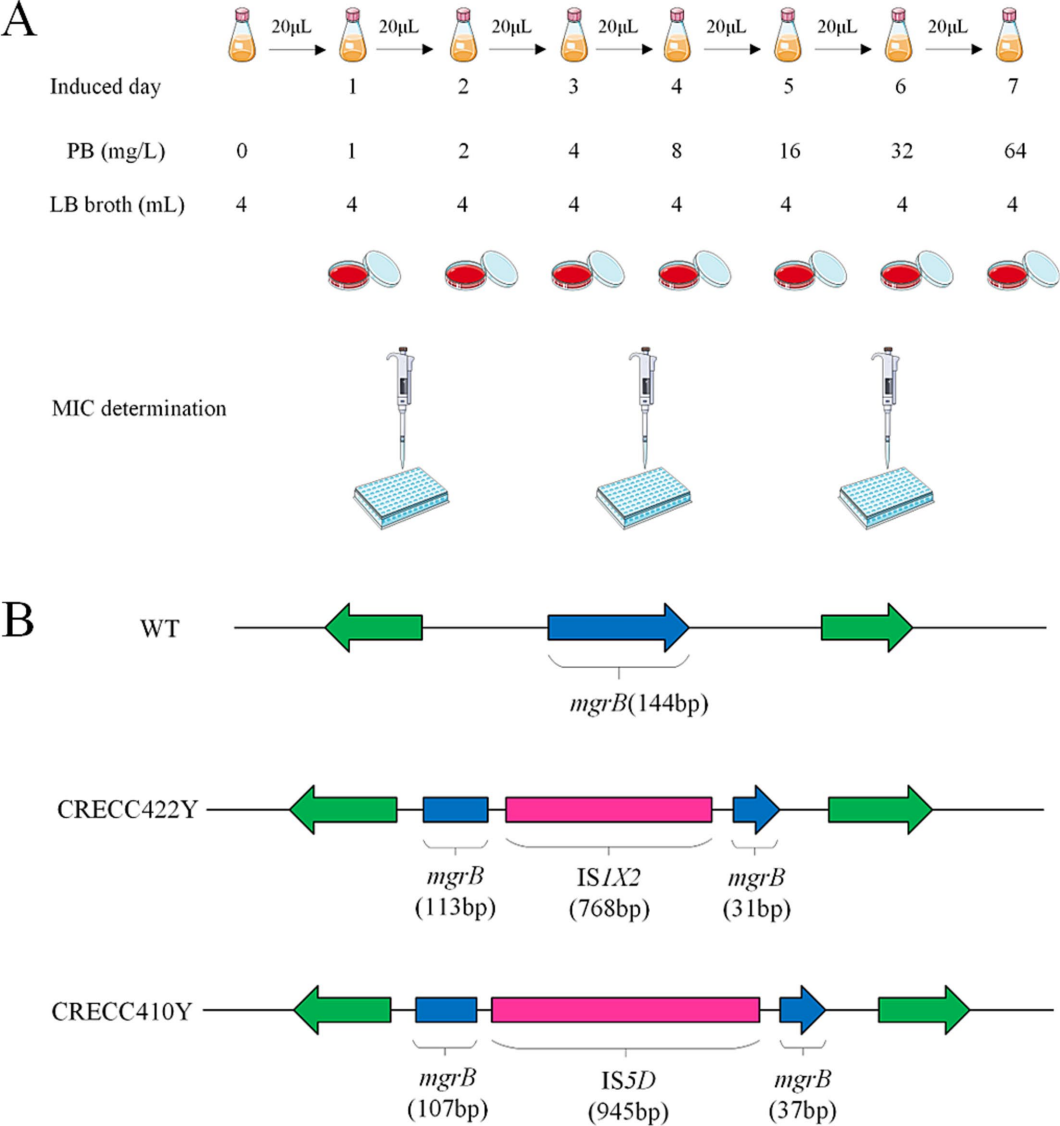
Experimental flow graph and analysis on insertional inactivated *mgrB* gene of *in vitro* polymyxin B inducted resistant variants. **(A)** Schematic diagram of polymyxin B induction experiment *in vitro*. **(B)** Insertional inactivation *mgrB* gene of carbapenem-resistant *Enterobacter cloacae* complex (CRECC) strains *in vitro*. Schematic representation of the *mgrB* gene *in vitro*-induced variants, CRECC422Y and CRECC410Y, derived from CRECC422 and CRECC410.

*In vitro* induction of PB was performed on 36 strains of low-level resistant CRECC (excluding CRECC405 and CRECC417) (Figure 4A; Table 3). After 7 days of continuous induction, the drug-resistant phenotype of 17 CRECC strains showed significant changes. These strains included *E. hormaechei* (*n* = 8), *E. asburiae* (*n* = 4), *E. kobei* (*n* = 2), *E. roggenkampii* (*n* = 1), *E. cloacae* (*n* = 1), and *E. mori* (*n* = 1). The MIC of PB was determined for these 17 induced CRECC strains using the microbroth dilution method. A significant increase in MIC values, ranging from 16 mg/L to >128 mg/L, was observed upon exposure to PB, as shown in Table 3.

**Table 3:** Polymyxin B (PB) resistance genes information and MICs of 36 CRECC strains.

Strain number	Pre-induction				Post-induction			
	*mgrB*	*phoP*	*phoQ*	PB MIC^a^ (mg/L)	*mgrB*	*phoP*	*phoQ*	PB MIC^a^ (mg/L)
CRECC66	WT	WT	WT	2	R47W	WT	WT	16
CRECC76	WT	WT	WT	4	WT	WT	Stop290	16
CRECC68	WT	WT	WT	4	WT	WT	A351T	32
CRECC410	WT	WT	WT	2	IS*5D*	WT	C423R	32
CRECC67	WT	WT	WT	2	WT	WT	E397A	64
CRECC72	WT	WT	WT	2	WT	WT	E232K	64
CRECC73	WT	WT	WT	4	Deletion	WT	WT	64
CRECC75	WT	WT	WT	4	WT	WT	E304V	64
CRECC422	WT	WT	WT	1	IS*1X2*	WT	WT	64
CRECC430	WT	Truncation	L9M; R69Q; Q141K; L168P; N169D; S193G	0.5	Deletion	Truncation	L9M; R69Q; Q141K; L168P; N169D; S193G	64
CRECC401	WT	WT	WT	2	WT	WT	Frameshift	>128
CRECC402	WT	WT	WT	2	WT	WT	WT	>128
CRECC412	WT	WT	Truncation	2	Deletion	WT	Truncation	>128
CRECC414	WT	WT	WT	4	A40P	WT	Frameshift	>128
CRECC416	WT	WT	WT	2	WT	WT	WT	>128
CRECC419	WT	WT	WT	2	Frameshift	WT	WT	>128
CRECC428	WT	WT	WT	1	WT	WT	Frameshift	>128

a Minimum inhibitory concentration.

### Molecular determinants of polymyxin resistance and mutation diversity of related genes

To investigate the molecular mechanisms underlying high-level drug resistance, we screened genes associated with the regulation of polymyxin resistance. Previous studies have identified the two-component system genes *phoP* and *phoQ*, as well as the negative regulatory gene *mgrB*, as crucial factors in polymyxin resistance (Doijad et al., 2023). Prior to exposure to PB, only CRECC430 and CRECC412 exhibited mutations (Table 3). In CRECC430, the *phoP* gene was truncated, and multiple amino acid substitutions were observed in *phoQ*. In CRECC412, the *phoQ* gene was truncated. The MICs of PB for pre-induction CRECC isolates ranged from 0.5 to 4 mg/L. After PB induction, the MICs of PB for 17 CRECC strains increased significantly, ranging from 16 to >128 mg/L (Table 3). The most frequently mutated gene was *phoQ* (58.8%, 10/17), with various mutations including amino acid substitutions (*n* = 5), frameshift mutations (*n* = 3), a nonsense mutation (*n* = 1), and gene truncations (*n* = 1) (Table 3). In contrast, no new mutations were found in the *phoP* gene after exposure to PB. The negative regulatory gene *mgrB* also exhibited multiple mutations, including deletions (*n* = 3), insertion sequences (*n* = 2), amino acid substitutions (*n* = 2), and a frameshift mutation (Table 3).

### Two insertion sequences have been identified for the first time in the *mgrB* of CRECC

Insertion sequence disruption of *mgrB* is a key mechanism for polymyxin resistance. We found two cases with insertion sequences in polymyxin-exposed strains, identified by IS Finder analysis as IS*1X2* (CRECC422Y) and IS*5D* (CRECC410Y) (Figure 4B). IS*1X2*, belonging to the IS*1* family, fragments the *mgrB* gene into 113 bp and 31 bp segments. IS*5D*, from the IS*5* family, is inserted after 107 bp of the *mgrB* gene. Notably, these insertion sequences are reported here for the first time in the *mgrB* gene of CRECC.

### Structural analysis of PhoQ protein at mutant sites

**Figure 5:**
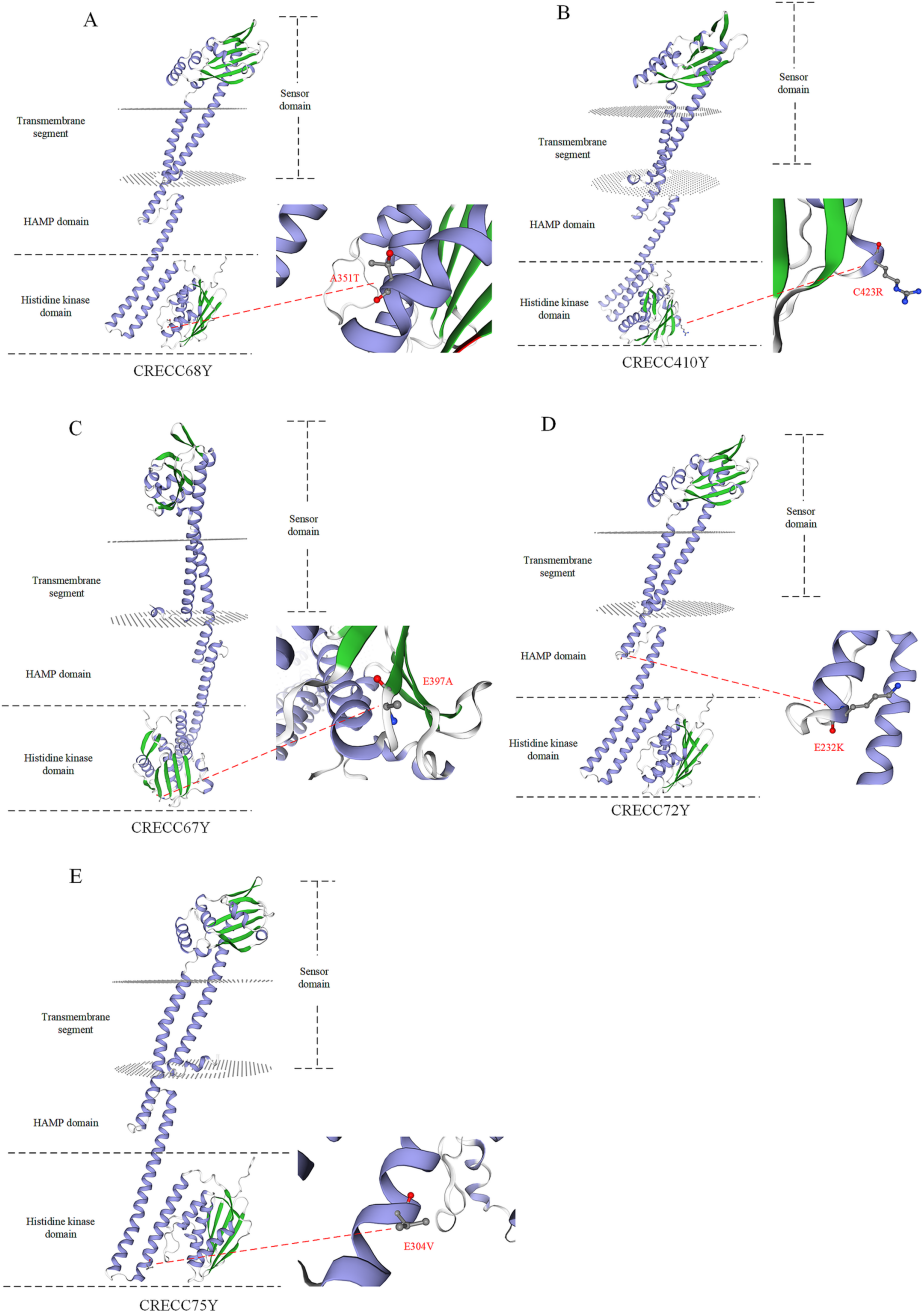
3D simulation snapshots of PhoQ protein in polymyxin B resistant mutant strains. **(A)** Resistant mutant strain CRECC68Y (A351T). **(B)** Resistant mutant strain CRECC410 (C423R). **(C)** Resistant mutant strain CRECC67Y (E397A). **(D)** Resistant mutant strain CRECC72Y (E232K). **(E)** Resistant mutant strain CRECC75Y (E304V). The alpha helix of the protein is represented by the blue band while beta type structure is depicted by the green band.

We identified several novel non-synonymous mutations in *phoQ* among polymyxin-resistant mutant strains (Figure 5; Table 3). To pinpoint these mutations, we used the AlphaFold sequencing scheme to model the structures of wild-type and mutant PhoQ dimers. The 3D structures of both wild-type and mutant PhoQ showed similar arrangements, covering the transmembrane region, periplasmic sensor domain, HAMP domain and histidine kinase region (Figure 5). The observed mutation sites in resistant mutants CRECC68Y (A351T), CRECC410Y (C423R), CRECC67Y (E397A), and CRECC75Y (E304V) were within the histidine kinase domain (Figures 5A–C,E). Secondary structure analysis revealed that all mutation sites, including CRECC68Y (A351T), CRECC410Y (C423R), CRECC72Y (E232K), and CRECC75Y (E304V), were within α helices (Figures 5A,B,D,E). Single point mutations in this region can affect domain energetics and their interactions, impacting polymyxin sensitivity (Mensa et al., 2021).

## Discussion

Clinical laboratories should focus on accurately identifying ECC species and providing improved guidance on carbapenem use. Additionally, polymyxin resistance in CRECC strains is increasingly common, with many resistant strains harboring the *mcr-9* gene (Kim et al., 2021; Wu et al., 2022; Yan et al., 2024). This paper discusses the specific effects of *mcr-9* carriage and overexpression.

This study found a significantly higher prevalence of the *mcr-9* gene in CRECC strains (26.3%) compared to other previous reports (Huang et al., 2020; Wang et al., 2020). Specifically, 9 strains were identified as *mcr-9.1* and only one as *mcr-9.2*, with *mcr-9.1* showing a more pronounced epidemic trend (Table 1). The prevalence of *mcr-9* in CRECC was notably higher than in CSECC, which is consistent with previous studies (Zhou et al., 2022). These findings highlight the need to be cautious about the potential impact of *mcr-9*. Despite this, the results indicated that both *mcr-9.1* and *mcr-9.2* had a very limited effect on PB resistance. This research represents the first exploration of how different subtypes of the *mcr-9* gene contribute to resistance. The *mcr-9*-mediated drug resistance may be linked to its expression level, as high expression of *mcr-9* is observed in polymyxin-resistant strains (Kieffer et al., 2019; Jiang et al., 2022). We introduced the plasmid pET-28a + *mcr-9.2* into the BL21(DE3) pLysS expression strain and induced high expression of *mcr-9.2* with IPTG. However, results showed that even with high *mcr-9.2* expression in BL21(DE3)pLysS+pET-28a + *mcr-9.2*(IPTG), there was only a two-fold increase in the MIC of PB compared to control strains (Table 2). Previous studies have demonstrated that the overexpression of *mcr-1* imposes a fitness cost on *E. coli* (Lu et al., 2022). To investigate whether *mcr-9* has similar effects in *E. cloacae*, we compared the growth of strains with high expression of *mcr-9* to control strains. No significant differences were observed between *E. coli* J68 and its control strain EC600, or *E. cloacae* CRECC68 and CRECC68-Δ*mcr-9.1*, suggesting that *mcr-9* alone does not significantly impact bacterial resistance or incur a fitness cost (Figure 2A). This lack of a fitness cost in *mcr-9*-positive strains might contribute to the widespread prevalence of the *mcr-9* gene in CRECC. However, IPTG-induced overexpression of *mcr-9* led to slower growth in strain BL21(DE3)pLysS+pET-28a + *mcr-9.2*(IPTG) compared to the control strain (Figure 2B). This suggests that elevated *mcr-9* expression imposes a fitness cost on BL21(DE3)pLysS+pET-28a + *mcr-9.2*(IPTG), though it does not confer high-level resistance to PB. Moreover, strain BL21(DE3)pLysS+pET-28a + *mcr-9.2* exhibited significantly increased membrane permeability in the presence of IPTG (Figure 3). We speculate that *mcr-9* overexpression may disrupt the bacterial outer membrane structure, increasing permeability and causing leakage of cell contents.

Exposure to PB has been shown to induce drug resistance, as demonstrated by reports of *Enterobacter* strains initially sensitive to PB evolving resistance during treatment and displaying a pronounced drug-resistant phenotype (Wu et al., 2023). In our *in vitro* studies, clinical CRECC strains with either PB sensitivity or low-level resistance were transformed to high-level resistance (PB MIC ≥16 mg/L) (Table 3). Sequence analysis of these genes after PB exposure revealed several CRECC strains with mutations including amino acid substitutions, frameshift mutations, premature terminations and partial deletions in the *phoQ* gene (Table 3). However, only one truncation was observed in the *phoP* gene, and it was not related to PB resistance (Table 3). The *phoQ* gene appears to play a more significant role than the *phoP* gene in the development of PB resistance in ECC. The *mgrB* gene plays a key role in acquired drug resistance (Mhaya et al., 2020; Yang et al., 2020). Among the CRECC strains, eight (22.2%, 8/36) showed alterations in the *mgrB* gene, including deletions (*n* = 3), insertion-induced inactivation (*n* = 2), amino acid substitutions (*n* = 2), and frameshift mutations (*n* = 1) (Table 3). We identified two novel mutations in the *mgrB* gene: a tryptophan substitution at position 47 in CRECC66 and a proline substitution at position 40 in CRECC414 (Table 3). Notably, insertion sequences were exclusively found in the *mgrB* gene, with IS*1X2* from the IS*1*-family and IS*5D* from the IS*5*-family disrupting the *mgrB* gene in CRECC422Y and CRECC410Y, respectively (Figure 4B). This represents the first report of two insertion sequences within the *mgrB* gene of ECC, highlighting the need for further surveillance to evaluate their potential widespread distribution. It is intriguing that strains CRECC402 and CRECC416 have no resistance mutations, and this observation requires further exploration of potential resistance mechanisms. Resistance could arise from regulatory changes rather than mutations directly associated with polymyxin resistance. For instance, alterations in the regulation of genes associated with the stress response, quorum sensing, or biofilm formation could indirectly confer resistance to polymyxins (Harms et al., 2016; Hwang and Kim, 2024).

Bacterial cells must continuously adapt to survive changing external environments. In bacteria and some lower eukaryotes, two-component systems connect extracellular stimuli with intracellular adaptive responses (Cheung et al., 2008). PhoQ, a transmembrane histidine kinase, is essential for sensing various environmental signals and is involved in the metabolic homeostasis and pathogenesis of many Gram-negative bacteria (Doijad et al., 2023). This study identified several novel mutations in the *phoQ* gene, prompting an evaluation of their potential impact on protein function. Mensa et al. demonstrated that E232A mutations exhibit diverse effects, revealing how allosteric coupling of domain equilibria is influenced by point substitutions (Mensa et al., 2021). Similarly, we identified the E232K mutation (CRECC72Y) in the HAMP domain which disrupts the balance between sensor and catalytic domains, significantly affecting ligand sensitivity of PhoQ as well as signal magnitude and direction (Figure 5D). This study highlights the prevalence of CRECC at the species level and elucidates the molecular mechanisms underlying high levels of PB resistance. However, while multiple mutations associated with high PB resistance were identified, further research is needed to fully understand their specific regulatory mechanisms.

## Conclusion

In conclusion, our study reveals that *E. hormaechei* is the predominant species, and the *bla*_NDM-1_ gene is a major contributor to carbapenem resistance in the clinical CRECC outbreak within this region. The prevalence of *mcr-9* in CRECC is higher than that in CSECC. However, both *mcr-9.1* and *mcr-9.2* genes are generally present in a silent carriage state in ECC.

Overexpression of *mcr-9* leads to a little increase in the MIC of PB, while also increasing membrane permeability and imposing fitness costs. It is worth emphasizing that *mcr-9* is not responsible for high levels of resistance in PB, but does not exclude possible resistance mutations.

Mutations in the two-component system genes *phoP*/*Q* and the negatively regulated gene *mgrB* are key mechanisms mediating high-level PB resistance, with *phoQ* showing a higher likelihood than *phoP* in conferring this resistance. The horizontal transfer potential of insertion sequences significantly contributes to polymyxin resistance, highlighting the need for prompt clinical intervention. Therefore, monitoring IS elements could be crucial in preventing the spread of PB resistance and reducing the risk of treatment failure.

## Data Availability

The genome sequence has been submitted to the National Center for Biotechnology Information, and the accession number is provided in the Supplementary Table S2. All primers used in this study are shown in the Supplementary Table S1.
