# Supplementary material for: Insertion sequences in mgrB and mutations in two-component system genes confer high polymyxin resistance to carbapenem-resistant Enterobacter cloacae complex strains
Source: Front Microbiol. 2025 Mar 17;16:1553148. doi: 10.3389/fmicb.2025.1553148 (PMC11955652; doi:10.3389/fmicb.2025.1553148)
Supplement: Supplementary file 1 [file Table_1.docx]

Table S1. Primers used in this work.

| Primer | Sequence (5’-3’) |
| --- | --- |
| *mcr-9* F： | CGGTACCGCTACCGCAATAT |
| *mcr-9* R： | ATAACAGCGAGACACCGGTT |
| *mcr-9*-5F: | GTGAAATCGCACACCTGGTAATTGATTC |
| *mcr-9*-5R: | GAAAAGTACAGGCATTAACACCTCCG |
| *mcr-9*-3F: | GTCTATAATCCTGACCGCGATTTGTTC |
| *mcr-9*-3R: | CATGTGCAGCTCCATCAGCAAAAG |
| *mcr-9*-linker: | CGGAGGTGTTA**ATGCCTGTACTTTTC**GTCTATAATCCTGACCGCGATTTGTTC |
| mcr-9-AprF: | CTTTTGCTGATGGAGCTGCACATGTAATGACCCCGAAGCAGGGTTATG |
| *mcr-9*-AprR: | GGAATAGGAACTTATGAGCTCAGCCAA |
| *mcr-9*-outF: | CTCTGATGAACGTTCCGCTACGCTG |
| *mcr-9*-outR: | CTCCTGCAGCTCACGGTAACTGATG |
| *mcr-9*-inF: | GCATTGCTTACCGTTTGCTCTCCGTG |
| *mcr-9*-inR: | GGATTTGCTGGTAAAGGCATTGGTATCACG |
| *mgrB*-Kobei F： | CACCTTAAGAAAAAATGCGTGCTAC |
| *mgrB*-Kobei R： | GGTCTTTAGACGGAGTGTGGA |
| *mgrB*-asburiae F： | CACCTTGAGAAAAAATGCGTATTAC |
| *mgrB*-asburiae R： | GGTTTTTAGACGGAGTGTGGAG |
| *phoP*-kobei F： | CATATTCTGGGAGAAAAGATGATGC |
| *phoP*-kobei R： | ATGGCGCAAAATCTGTCTCATTTAG |
| *phoP*-asburiae F： | ATTATTTAGGGAGAAGAGATGATGC |
| *phoP*-asburiae R： | GTGGCGTAAAATCCCTCTCATT |
| *phoQ*-kobei 1F： | TCGCATTTTTCACATAACGGGTTAA |
| *phoQ*-kobei 1R： | GCGAGCTTGAGGAACACCATC |
| *phoQ*-kobei 2F： | CTTTTCAGCAGGCGATTGAGAT |
| *phoQ*-kobei 2R： | TACCTGTTTGAATTACGCTAAATGAG |
| *phoQ*-asburiae 1F： | TCGCATTTTTCACATAACGGGTTAA |
| *phoQ*-asburiae 1R： | GTGAGCTGGAAGAACATCATCG |
| *phoQ*-asburiae 2F： | CTTTTCAGCAGGCGGTTGAGG |
| *phoQ*-asburiae 2R： | TACCTGTTCGAATTACGCTAAATGAG |
